# Examining school-based hygiene facilities: a quantitative assessment in a Ghanaian municipality

**DOI:** 10.1186/s12889-018-5491-9

**Published:** 2018-05-02

**Authors:** Emmanuel Appiah-Brempong, Muriel J. Harris, Samuel Newton, Gabriel Gulis

**Affiliations:** 1 Department of Health Promotion and Education, Kwame Nkrumah University of Science and Technology (KNUST), Kumasi, Ghana; 2 Department of Health Promotion and Behavioral Sciences, University of Louisville, Louisville, USA; 3 Department of Global and International Health, Kwame Nkrumah University of Science and Technology (KNUST), Kumasi, Ghana; 4 Unit for Health Promotion Research, University of Southern Denmark (SDU), Esbjerg, Denmark

**Keywords:** School, Water, Hygiene, Functional, Facilities

## Abstract

**Background:**

The crucial role of adequate water, sanitation and hygiene (WASH) facilities in influencing children’s handwashing behaviour is widely reported. Report from UNICEF indicates a dearth of adequate data on WASH facilities in schools, especially in the developing world. This study sought to contribute to building the evidence-base on school hygiene facilities in Ghana. The study further explored for possible associations and differences between key variables within the context of school water, sanitation and hygiene.

**Methods:**

Data was collected from 37 junior high schools using an observational checklist. Methods of data analysis included a Scalogram model, Fisher’s exact test, and a Student’s *t*-test.

**Results:**

Results of the study showed a facility deficiency in many schools: 33% of schools had students washing their hands in a shared receptacle (bowl), 24% had students using a single cotton towel to dry hands after handwashing, and only 16% of schools had a functional water facility. Furthermore, results of a proportion test indicated that 83% of schools which had functional water facilities also had functional handwashing stations. On the other hand, only 3% of schools which had functional water facilities also had a functional handwashing stations. A test of difference in the proportions of the two sets of schools showed a statistically significant difference (*p* < 0.001).

In addition, 40% of schools which had financial provisions for water supply also had functional handwashing stations. On the other hand, only 7% of schools which had financial provisions for water supply also had functional handwashing stations. There was a statistically significant difference in the proportions of the two sets of schools (*p* = 0.02).

**Conclusion:**

We conclude that it is essential to have a financial provision for water supply in schools as this can potentially influence the existence of a handwashing station in a school. An intervention by government, educational authorities and civil society organisations towards enabling schools in low resource areas to have a sustainable budgetary allocation for WASH facilities would be timely.

## Background

Infectious diseases continue to claim many lives especially among children. Though the developed world is not exempted from this phenomenon, the developing world is often hit the hardest. For example, diarrhoeal diseases have been listed as one of four leading causes of early death in all sub-Saharan African countries [1]. In the year 2015, approximately 1400 children lost their lives each day as a result of diarrhoea [2]. Preventing the spread of infectious agents in schools is a good way towards minimizing the infectious disease burden among children. The provision of adequate water, sanitation and hygiene (WASH) facilities in schools is crucial in ensuring the adoption and maintenance of safe sanitation and hygiene practices among school children.

The available evidence suggests that schools with better hygienic conditions tend to have less problems with disease causing organisms [3]. In the light of this, WASH in schools deserves increased attention both at the global and national levels. A laudable expression in the UN Sustainable Development Goals (SDGs) is the recognition of WASH in Schools (WinS) in Goals 4 and 6. However, such global expressions ought to be translated into national and local policies and actions in order to improve WinS substantially.

The crucial role of adequate WASH facilities towards influencing children’s handwashing behaviour is widely known [4, 5]. A functional handwashing station in a school makes it possible for children to adopt the practice of handwashing with soap (HWWS). In spite of this importance, national monitoring systems for WASH which are intended to report on the availability and state of handwashing facilities in schools is generally weak especially in developing countries. Few developing countries have reliable data on hygiene facilities in schools, of which Ghana is no exception. According to UNICEF, only about half of its programme countries are able to report on WASH facilities in schools [6]. The dearth of reliable data on the functionality of WASH facilities in schools is worrisome, as such data is required for good programme design and management [6].

There is a paucity of studies examining hand hygiene facilities in African schools. In Ghana, Steiner-Asiedu et al. as well as Monney et al. have attempted to describe the available handwashing facilities in a sample of schools [7, 8]. However, no study was identified to have quantitatively explored the associations or variance between a functional water facility, financial provision for water supply, and existence of a handwashing station. The present study sought to employ a more robust quantitative approach to generate evidence on the existence and functionality of hygiene facilities within a representative sample of basic schools in a Ghanaian municipality. Precisely, we sought to test the following null hypotheses:

1. **Ho:** With regards to the existence of a functional handwashing station, the proportion of schools having a financial provision for water supply will not differ from the proportion of schools lacking a financial provision for water supply.

2. **Ho:** With regards to the existence of a functional handwashing station, the proportion of schools having a functional water facility will not differ from the proportion of schools lacking a functional water facility.

## Methods

### Approach and design

The methodological approach adopted for the study was quantitative. A study seeking to objectively assess the existence, proportions and relationships between existing facilities is better approached from a positivist perspective, and hence the choice of a quantitative methodology. A cross-sectional design was employed for this assessment, and so study variables were assessed at only one point in time.

### Study population and sample size

The study population was made up of all public junior high schools within the Ejisu-Juaben Municipal Education Directorate. In all, there were 80 public junior high schools organised within 10 educational circuits. The list of schools was obtained from the Municipal Education Directorate. A representative sample of 37 schools was derived using two complementary formulas by Yamane and the Pennsylvania State University [9, 10].

### Sampling technique

In a bid to ensure that each school in all 10 educational circuits had an equal chance of being a part of the study, and in a manner that enhances the external validity of study results, a *stratified random sampling* technique was employed. With this technique, educational circuits constituted the strata, while schools constituted the sample units. In line with the above technique, a *proportionate stratification* approach was used [11]. This approach ensured that the sample size (i.e. number of schools required from each educational circuit) of each stratum (i.e. an educational circuit) was proportional to the population size of the stratum being considered [12]. Table 1 shows the estimated number of schools from each educational circuit.

**Table 1:** School Selection

ID	EDUCATIONAL CIRCUIT	POPULATION SIZE OF CIRCUIT	ESTIMATED NO. OF SCHOOLS REQUIRED
A	Achinakrom	6	3
B	Bomfa	8	4
C	Ejisu	11	5
D	Fumesua	5	2
E	Kubease	8	4
F	Kwaso	9	4
G	New Koforidua	6	3
H	Ofoase	5	2
I	Juaben	14	6
J	Tikrom	8	4
TOTAL		80	37

Source: Developed based on a list obtained from the EJM Education Directorate, 2016

### Description of data collection procedures and tools

Data was collected from March 2016 to July 2016, spanning a period of five calendar months. A checklist adapted from UNICEF and Moore et al. aided the environmental audit of schools in order to obtain first hand data on existing facilities [6, 13]. A decision on which facilities to target was guided by a WASH in schools monitoring package developed by UNICEF [6]. The observation tool was pretested in a junior high school located in a municipality which is contiguous to the geographic scope of this study (student enrolment, *n* = 256), and was subsequently fine-tuned. For instance, after the pretest it became necessary to include an item that enabled the observation of a functional toilet facility within schools. This was essential due to the complementarity of the concepts of hygiene and sanitation. Where required, clarifications on facilities were sought from the head teacher or an authorized representative of the school. The checklist makes use of dichotomous questions and assesses the school environment based on predetermined parameters, including: accessibility and source of water point, existence of handwashing stations, functionality of toilet facility, and accessibility to the toilet facility.

Data was collected by the lead researcher with assistance from trained field enumerators. Field enumerators were given a 3-h training by the lead researcher who has substantial experience in observational studies. All field enumerators were university graduates serving as teaching and research assistants in a public university.

### Data analysis

Descriptive statistical analysis was used in describing the existing facilities for handwashing in schools. For profiling the existing WASH facilities in participating schools, a scalogram model was used. In exploring for possible relationships between key variables, and differences in proportions of variables, statistical tests used were the Student’s *t*-test, Fisher’s exact test, and a two-sample proportion test. Data was analyzed using STATA *version 14.0* (*STATA Corp., College Station, Texas*).

### Ethical considerations

The research protocol was reviewed and subsequently granted clearance by the Committee on Human Research, Publications and Ethics (CHRPE) of the Kwame Nkrumah University of Science and Technology, Ghana. In addition, an approval to conduct the research was obtained from the Ejisu-Juaben Municipal Education Directorate of the Ghana Education Service, a regulatory body of all basic schools within the study area.

## Results

### Characteristics of schools, and distribution of facilities

Observations of hygiene facilities occurred in all 37 schools. All participating schools were public, and none was a single-sex school. The mean student enrolment was 153 (SD = 81.9). The minimum student enrolment was 46 while the maximum was 394. Table 2 presents summary statistics on the proportions of schools with specific facilities.

**Table 2:** Proportion of Schools with Specific Facilities

S/N	Item	Number of Schools	Percentage (%)
A	Functional^b^ water point/facility	6	16
B	Functional^b^ water reservoir	4	11
C	Soap	25	68
D	Tap bucket (veronica bucket)	13	36
E	Shared/communal ^a^HW receptacle	12	33
F	Receptacle (for waste water)	11	31
G	Paper/tissue towel	0	0
H	Shared cotton towel/napkin	9	24
I	Functional handwashing station	6	16
J	Functional toilet facility	21	56
Financial Provision for HW Facilities			
K	Financial Provision for facilities	10	27
L	Source of funds		
	o Capitation grant	1	6
	o Internally Generated	9	94

Source: Field Survey, 2017 ^a^HW-Handwashing ^b^Facility was usable on the day of observation

Soap was the most common item observed in participating schools. Sixty-eight percent (68%) of schools had soap available for use. Out of the number of schools having soap (*n* = 25), 4% had a liquid soap, while the remaining schools had a solid soap (96%). Sixteen percent (16%) of schools had a functional water facility within its compound. Most of these facilities were boreholes (in 83% of schools), and 17% had a stand-pipe (tap-water).

Of the 37 participating schools, 6 (16%) had a functional handwashing station. The average number of functional handwashing stations was 2 (SD = 0.98). Only 19% of schools had a handwashing facility attached to a toilet facility. Also, 23% of schools had a separate handwashing station for teachers. Also, 33% of schools had students washing their hands in a shared receptacle (bowl), 24% had students using a single cotton towel to dry hands after handwashing, and none of the schools had a paper towel displayed for hand drying.

With regards to functional toilet facilities, 56% had this within the school compound. The types of toilet facility observed were the WC (6%), VIP (6%) and the Simple Pit Latrine (88%). The mean number of cubicles within a toilet facility was 4.9 (SD = 2.6). All schools had a separate section of the toilet facility for males and females. Also, 75% of schools had a separate section designated for teachers.

A financial provision for running handwashing stations reportedly existed in 27% of schools. Two main streams of revenue for sustaining a WinS were identified. These were the “capitation grant” from central government, and the internally generated funds (IGF). Most schools (94%) depend largely on the IGF for maintaining WASH facilities. Inflows to the IGF were identified to be from the sporadic donations from the Parents and Teachers Association (PTA), as well as the weekly fundraising sessions held during religious activities in schools. With regards to the adequacy of funds for maintaining WASH facilities, all schools (*n* = 21) described their financial resources as inadequate.

### A profile of facilities in schools

The observed handwashing (HW) and related facilities in participating schools are profiled with the aid of a scalogram model presented as Table 3. With reference to Table 3, columns 1 and 2 present a list of all sampled schools with their respective student enrolment figures. The second row presents the facilities assessed in this study. Corresponding to the facilities are codes indicating whether or not a particular facility exists in a particular school. Thus, code “1” means a facility exist, while code “0” means otherwise. At the base of the matrix are the corresponding weights of each of the facilities calculated by dividing the assumed centrality by the total number of facilities. Columns 11 and 12 shows the total number of facilities in each school as well as the centrality indices. Centrality index is a sum of the weights of all facilities which exist in a particular school.

**Table 3:** A Scalogram Model on Schools and Functional Facilities

School Identifier	Student Enrolment M(SD) = 153(81.9)	Existing Functional Facilities								Total no. of facilities	Centrality Indices
		Water Point	Hand-washing Station	Water Reservoir	Tap Bucket	Soap	Receptacle (waste water)	Toilet	Budget-Water Supply		
0037	309	1	1	1	1	1	0	1	1	7	84.8
0027	198	1	1	1	1	1	0	1	0	6	74.8
0031	84	1	1	0	1	1	1	1	1	7	68.8
0023	178	1	1	0	1	1	0	1	1	6	59.8
0036	177	0	1	0	1	1	1	1	1	6	52.1
0028	201	0	0	1	0	1	1	1	0	4	42.7
0003	99	1	0	0	1	1	0	0	1	4	38.4
0015	46	1	1	0	0	1	0	0	0	3	37.4
0026	144	0	0	0	1	1	1	1	1	5	35.4
0029	258	0	0	1	0	1	0	1	0	3	33.7
0010	108	0	0	0	1	1	1	1	0	4	24.7
0001	119	0	0	0	0	1	1	0	1	3	23
0011	142	0	0	0	1	1	1	0	0	3	20.7
0025	103	0	0	0	0	1	0	1	1	3	18.7
0006	157	0	0	0	0	1	1	1	0	3	17.7
0008	98	0	0	0	1	0	0	0	1	2	17.7
0030	92	0	0	0	0	1	1	1	0	3	17.7
0034	180	0	0	0	0	1	1	1	0	3	17.7
0020	112	0	0	0	1	1	0	1	0	3	16.4
0032	50	0	0	0	0	1	1	0	0	2	13
0035	75	0	0	0	1	0	0	1	0	2	12.4
0024	196	0	0	0	1	1	0	0	0	2	11.7
0004	100	0	0	0	0	0	0	0	1	1	10
0005	205	0	0	0	0	1	0	1	0	2	8.7
0017	227	0	0	0	0	1	0	1	0	2	8.7
0022	106	0	0	0	0	1	0	1	0	2	8.7
0007	296	0	0	0	0	0	0	1	0	1	4.7
0018	163	0	0	0	0	0	0	1	0	1	4.7
0033	334	0	0	0	0	0	0	1	0	1	4.7
0014	48	0	0	0	0	1	0	0	0	1	4.0
0021	134	0	0	0	0	1	0	0	0	1	4.0
0002	98	0	0	0	0	0	0	0	0	0	0.0
0009	72	0	0	0	0	0	0	0	0	0	0.0
0012	119	0	0	0	0	0	0	0	0	0	0.0
0013	119	0	0	0	0	0	0	0	0	0	0.0
0016	394	0	0	0	0	0	0	0	0	0	0.0
0019	130	0	0	0	0	0	0	0	0	0	0.0
Total number of facilities		6	6	4	13	25	11	21	10		
Assumed centrality		100	100	100	100	100	100	100	100		
Weight		16.7	16.7	25	7.7	4	9.0	4.7	10		

Source: Author’s Construct, 2017

From Table 3, school with identifier 0037 had the highest centrality index (CI = 84.80) by having nearly all observed facilities in school. On the other hand, schools with the lowest CI include 0002, 0009, 0012, 0013, 0016, and 0019 (all with a CI = 0.0), depicting the non-existence of at least one of the facilities for which observations were made.

### Differences in proportions of facilities in schools

Differences in proportions, and differences in means of group variables were explored and the results are presented in Table 4. Results of an independent samples *t*-test shows a statistically significant difference in mean student enrolment of schools which had a functional toilet facility and schools which did not a functional toilet facility [*t*(35) = − 2.06, *p* = .04]: Schools which had a functional toilet facility were observed to have a higher mean enrolment fig. [M = 177(SD = 76)], when compared with schools which did not have this facility [(M = 123(SD = 82)]. On the contrary, there was no statistically significant difference in the mean student enrolment of schools where a functional handwashing station existed and schools where it did not exist [*t*(35) = − 0.38, *p* = .69].

**Table 4:** Differences in Proportions and Means

Student Enrolment		
Response Variables	*p*-value (α < 0.05)	Mean(SD)
Functional Toilet Facility	0.04	
- Exist		177(76)
- Does not exist		123(82)
Functional HWS*	0.69	
- Exist		165(92)
- Does not exist		150(81)
	Functional HWS	
Explanatory Variables	*p,* Fisher’s Exact	Cramer’s V
Functional Water facility	0.00	0.80
Functional Toilet facility	0.16	–
Financial Provision for WS^+^	0.03	0.39
Two-Sample Proportion Test		
	*z* (*p*-value)	Proportion (95% CI)
Functional Water facility	−4.87 (0.00)	
- Within School		0.83 (0.54–1.13)
- Outside School		0.03 (−0.03–0.10)
Financial Provision for water supply	−2.38 (0.02)	
- Exist		0.40 (0.10–0 .70)
- Does not exist		0.07 (− 0.02–0.17)

*HWS-Handwashing station ^+^WS-Water supply

To determine the possible associations existing between explanatory variables (i.e. functional water facility, functional toilet facility, existence of a financial provision for water supply), and a response variable ‘functional handwashing station’, results of a Fisher’s exact test (two-tailed) indicated a statistically significant association between ‘functional handwashing station’ and ‘functional water facility’ (*p* < .001; Fisher’s exact), and also ‘financial provision for water supply’ (*p* = .03; Fisher’s exact), but not ‘functional toilet facility’ (*p* = .16; Fisher’s exact).

In a bid to further examine the differences in proportions of each of the pairs of categories of explanatory variables (where a statistically significant *p*-value was generated), results of a two-sample proportion test (two-tailed) indicated that 83% of schools which had functional water facilities also had functional handwashing stations. On the other hand, only 3% of schools which had functional water facilities also had functional handwashing stations. A test of difference in the proportions of the two sets of schools showed a statistically significant difference (*z* = − 4.87, *p* < 0.001).

In addition, 40% of schools which had financial provisions for water supply also had functional handwashing stations. On the other hand, only 7% of schools which had financial provisions for water supply also had functional handwashing stations. There was a statistically significant difference in the proportions of the two sets of schools (*z* = − 2.38, *p* = 0.02).

## Discussion

### Distribution of facilities in schools

The study has shown that most schools had soap available to students (68%). Also, few schools had a functional water facility (16%). Similarly, few schools had a functional handwashing station (16%), which leaves much to be desired, considering the fact that 21% of schools in the developing world have handwashing facilities [14]. Furthermore, a large number of students wash their hands in a shared receptacle and also dry their hands with a common towel. More so, only half of the number of schools have a functional toilet facility within the schools’ premises. In such a situation, students could potentially resort to public toilet facilities, private homes or open defecation. It is however worth noting that the existence of a toilet facility in a school does not automatically guarantee its usage. Factors including facility age, and facility type could influence facility usage [15]. In this study, a simple pit latrine is identified to be the most common toilet facility in participating schools (88%). This condition obviously raises concern since a pit latrine is an unimproved toilet type, and hence not recommended for a school setting [5].

The availability of soap in schools is crucial to the promotion of school-based handwashing with soap (HWWS). However, it is well understood that the mere availability of soap does not imply the existence of a handwashing station, as additional facilities are required. Though the results show that most schools had soap available, it is also evident that few schools had a functional handwashing station, and therefore the use of soap (which was mostly found in front of offices of teachers) could have been used for other purposes such as washing teachers’ dishes after meals. The commonality of soap is consistent with the result of a study conducted in Ghana which showed that 96% of study settings had soap available, but were used for other purposes apart from handwashing (e.g. washing dishes) [16]; and with another which reported that 83% of a sample of schools in Ghana had soap available [8]. The result is however in contrast to that of a study conducted in Senegal which reported that only 10% of participating schools had soap available to students [17].

Furthermore, the situation in which only 19% of schools had a handwashing facility attached to (or located within the premises) of a toilet facility appears worrisome and inconsistent with existing guidelines for setting up WASH facilities in schools [5]. It is common knowledge that the proximity of a handwashing facility to a toilet facility can potentially influence adherence to HWWS after toilet use.

The situation in which a large number of schools use a shared receptacle for students’ handwashing, and a shared cotton towel for students’ hand drying leaves much to be desired. In a country where infectious diseases continue to claim many lives, the implications of this practice on cross-infections is a matter of concern. In a recent study conducted in Ghanaian preschools, 8 different bacteria, 2 different parasites and a fungus were observed in water samples collected from a shared receptacle used by school children for handwashing [18].

The gloomy situation described above could potentially be explained in part by the WASH facility deficiency which appears to be characteristic of many schools in the municipality. The result of the scalogram analysis points to a situation where many schools are constrained in terms of basic facilities such as a functional water facility, a functional handwashing station, paper towels, and a financial provision for WASH. An observation of the centrality indices from the scalogram matrix shows that some schools (16%) have as low as 0.0 index indicating the non-existence of any of the observed WASH facilities. The WASH facility deficiency characterising participating schools is similar to a situation reported in Malawi where only 33% of handwashing facilities in schools were functional, and no school had soap available for handwashing [19]. An intervention by government and civil society organisations in addressing the situation will be timely.

### Differences in proportions of handwashing facilities

With respect to functional toilet facilities in schools, the mean student enrolment was higher for schools which had this facility when compared with schools which lacked this facility. The higher student enrolment figures observed among schools with a functional toilet facility could suggest the possibility of a facility-driven selection of schools by parents or guardians. However, it is beyond the purview of this present study to draw such conclusions since additional studies may be required to draw a conclusion. The existence of a functional toilet facility was however not associated with the existence of a functional handwashing station, which is an issue of concern considering the crucial complementarity of a handwashing station and a toilet facility [5].

Furthermore, a greater proportion of schools which had a functional water facility also had a functional handwashing station. The existence of a functional water facility in a school is crucial to the setting up of a functional handwashing station. Among others, this facilitates the provision of adequate running water which has been identified as key to the practice of proper handwashing with soap [20, 21].

Similarly, a greater proportion of schools which had a financial provision for water supply also had a functional handwashing station. To ensure the sustainability of functional handwashing stations in schools, the role of a budgetary allocation is imperative, and the result of this study has shown that a financial provision is essential to the existence of a functional handwashing station in schools. Recognizing the crucial role of a financial provision, it becomes a concern that few schools reported the existence of this scheme (27%). This result is consistent with that of a study conducted in Nicaragua where 95% of schools did not have a budgetary allocation for the provision of an essential WASH facility as soap [22].

### Limitation

The generalization of results of this study is limited to settings which have socio-economic characteristics similar to that of the geographic scope of this study.

## Conclusion

The importance of having an enabling environment to enhance the adoption of healthful behaviours is widely known among the health promotion fraternity. Ensuring the existence of adequate hygiene facilities in schools is a good way towards creating an environment which enables the adoption and maintenance of a safe hygiene behaviour among school children. The evidence generated by this study suggests a hygiene facility deficit across a range of schools.

Also, results of this study indicate that there is not enough evidence to accept the two null hypotheses formulated. Thus, schools with a financial provision for water supply tend to have a functional handwashing station. Similarly, schools with a functional water facility within its compound tend to have a functional handwashing station. This implies that concerted efforts by government, educational authorities and civil society organisations towards assisting schools especially in low resource areas to have a sustainable budgetary allocation for WASH facilities will be imperative.

## Abbreviations

CHRPE: Committee on Human Research Publication and Ethics
CI: Centrality Index
HA: Alternative Hypothesis
Ho: Null Hypothesis
HW: Handwashing
HWWS: Handwashing with Soap
M: Mean
SD: Standard Deviation
UNICEF: United Nations Children’s Fund
WASH: Water, Sanitation and Hygiene
WinS: WASH in School
